# Are genetic drift and stem cell adherence in laboratory culture issues for cultivated meat production?

**DOI:** 10.3389/fnut.2023.1189664

**Published:** 2023-08-28

**Authors:** Manuel Jaime-Rodríguez, Ana Laura Cadena-Hernández, Lorena Denisee Rosales-Valencia, Juan Miguel Padilla-Sánchez, Rocio Alejandra Chavez-Santoscoy

**Affiliations:** Tecnologico de Monterrey, School of Engineering and Sciences Monterrey, Monterrey, Mexico

**Keywords:** genetic drift, cultivated meat, mesenchymal stem cells, quality control, passages, stemness

## Abstract

Mesenchymal stem cell-based cultivated meat is a promising solution to the ecological and ethical problems posed by traditional meat production, since it exhibits a protein content and composition that is more comparable to original meat proteins than any other source of cultivated meat products, including plants, bacteria, and fungi. Nonetheless, the nature and laboratory behavior of mesenchymal stem cells pose two significant challenges for large-scale production: genetic drift and adherent growth in culture. Culture conditions used in the laboratory expose the cells to a selective pressure that causes genetic drift, which may give rise to oncogene activation and the loss of “stemness.” This is why genetic and functional analysis of the cells during culture is required to determine the maximum number of passages within the laboratory where no significant mutations or loss of function are detected. Moreover, the adherent growth of mesenchymal stem cells can be an obstacle for their large-scale production since volume to surface ratio is limited for high volume containers. Multi-tray systems, roller bottles, and microcarriers have been proposed as potential solutions to scale-up the production of adherent cells required for cultivated meat. The most promising solutions for the safety problems and large-scale obstacles for cultivated meat production are the determination of a limit number of passages based on a genetic analysis and the use of microcarriers from edible materials to maximize the volume to surface proportion and decrease the downstream operations needed for cultivated meat production.

## Introduction

According to the United Nations (1), the world population will reach almost 10 billion people in 2050. This correlates directly to an increase in meat consumption, which is projected to grow 14% from 2018 to 2030 (2).

Traditional meat production methods have been correlated with various problems in soil and water consumption and pollution, loss of habitat for multiple species, loss of biodiversity, and significant greenhouse gas emissions (3). Current meat production is performed following these stages (4): cattle breeding, feedlot, transportation, slaughter, processing, and supplying.

This process leads to the copious production of greenhouse gasses, not just by the livestock (5), but also by the production of its feed, as well as the machines and vehicles involved in the transport and processing of the meat (6). To mention a few, methane is produced as a product of ruminant metabolism (7), nitrous oxide is generated from the fertilizers that are used to produce grain for cattle feedlot (8), and carbon dioxide is a result of the fuel combustion used for animal transportation, as well as their food and other inputs that are used throughout the meat production process (9).

In round figures, the production of a metric ton of meat requires 100 Gigajoules of energy, 5,000 cubic meters of water, 20,000 square meters of earth and 20,000 kilograms of greenhouse gasses (10). An optimization of the traditional meat production process is needed to reduce its weighty environmental impact. Additionally, the implementation of an alternative production process would aid in the fulfillment of much-needed climate change policies.

Cultivated meat is one of the proposed alternatives to the traditional meat production process. This, owing to the optimization of resource consumption in its production, as well as requiring less surface area than the traditional process. As of today, there are four main types of recognized cultivated meat products (11), classified by source: animal, plant, fungi, and bacteria.

Of the four sources, fungi (12) and plant-based meat have developed more into commercial products available to the customer (13) at a friendly cost and best matching the organoleptic properties of meat (14). However, the overall nutritional composition of plant and fungi-based meat is different to that of animal meat (15) in terms of protein concentration and composition (16), offering an opportunity to animal cell-based cultivated meat.

As for bacterial cultivated meat production, it is restricted to the production of three-dimensional cellulose or calcite structures that require the addition of plant, fungi or animal protein to produce the complete meat product (17). This property has been widely substituted by three-dimensional food printing due to its precision and versatility (18).

Although animal cell-based cultivated meat best resembles traditional meat, the production process faces limitations with industrial scaling-up and changes in genetic expression and physiological properties of the cells during laboratory culture that can affect process reproducibility and product safety; both subjects will be widely explained through this review.

## Mesenchymal stem cell-based cultivated meat production

There are two fundamental techniques used for the generation of cultivated meat (19): the self-organizing technique and the scaffold-based technique (20). The self-organizing technique requires using an explant from the muscle of a donor animal, followed by its proliferation in a nutrient medium, while the scaffold-based technique involves the use of suitable stem cells, which are then attached to a scaffold (three-dimensional matrix) or a carrier and grown in a bioreactor with culture medium (21).

The initial efforts to produce cultivated meat were based on the tissue explant technique. The first attempt was performed by NASA by culturing muscular structure of golden fish under laboratory conditions (22), while the second one was a research project that produced a 33,000-British-pound hamburger meat based on beef muscular explant (23).

Both cultivated meat products were produced by the removal of a fraction of tissue from an animal followed by its maintenance in culture media to preserve the tissue alive and growing (24). However, this technique was found unsuitable for large-scale meat production due to the explants not surviving more than 2 months in laboratory culture (25), as well as having a marginal growth due to a lack of gas and nutrient diffusion (26). Normally, organ nutrient diffusion is handled by the vascular system, which has not yet been emulated under laboratory conditions (27).

Stratospheric costs, low production efficiency and the large-scale limitations are the main reasons why explant technique has been completely substituted by the scaffold-based technique under laboratory conditions, since it provides a higher efficiency to produce cells while having a lower cost.

Compared to explant-based culture, the growth of individually separated cells eases the nutrient and gas diffusion, both of which are fundamental to culture cells on a large scale. This technique consists mainly of stem cells that can be reproduced in the laboratory and differentiated into fibroblasts to constitute the building blocks of cultivated meat (21).

The four types of stem cells that are used for cultivated meat production processes are embryonic stem cells (28), induced pluripotent stem cells (23), satellite cells (29), and mesenchymal stem cells (30).

**Figure 1:**
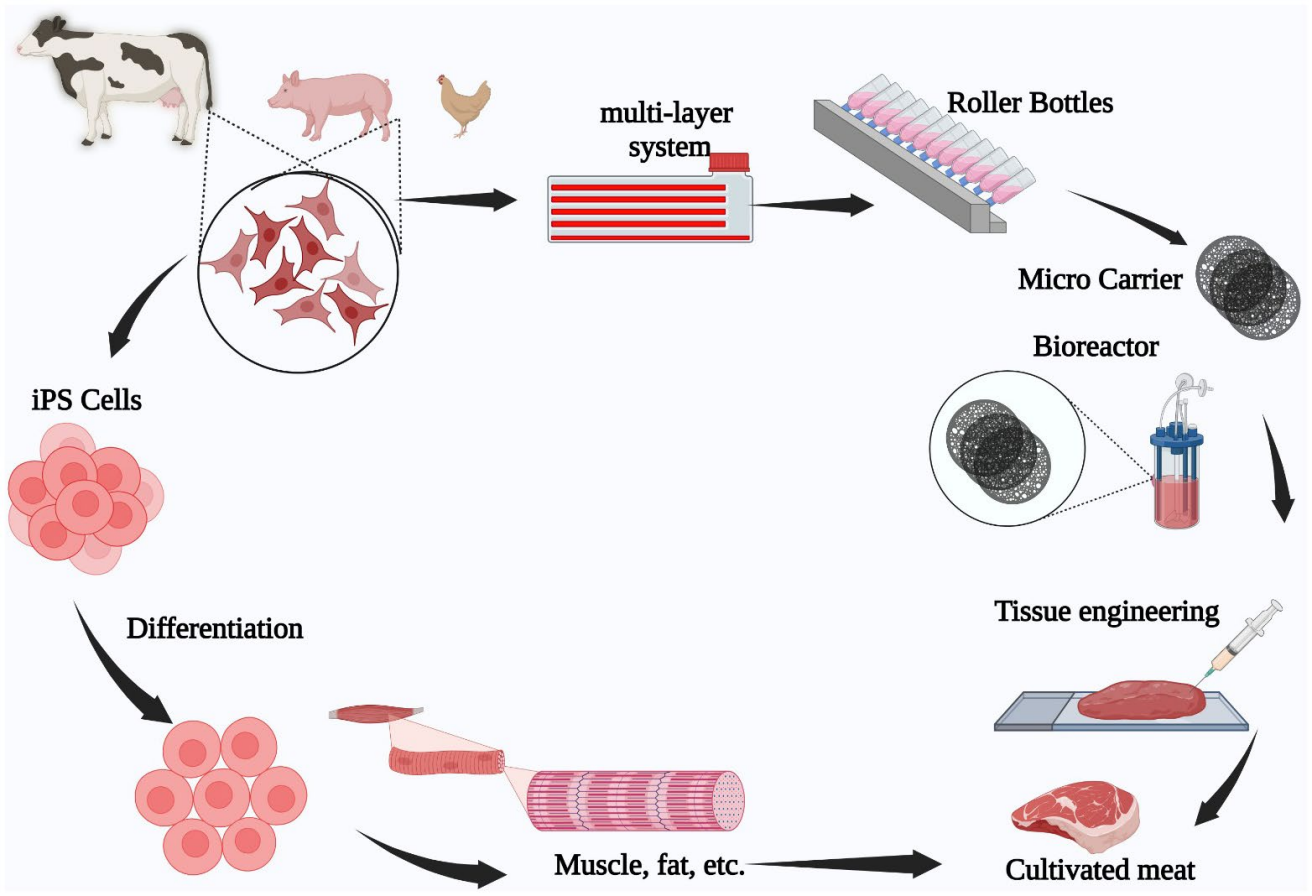
Animal stem cell-based cultivated meat production process. Tissue is extracted from an animal (pig, chicken, beef), following which, stem cells are isolated and grown in a laboratory to expand them. Finally, the cells are differentiated into muscle or fat tissue to engineer the final meat product.

The aforementioned cell types are cultured so they reproduce into hundreds of millions of cells, once obtained from cattle animals such as chickens, pigs, or cows. Afterwards, they are differentiated into muscular tissue (31) to be assembled in muscular fibers and integrated to form meat products fully produced within a laboratory (32), as shown in Figure 1.

Mesenchymal stem cells are considered suitable meat constituents due to their ubiquity and ease of collection, since each connective tissue may be a source of mesenchymal stem cells. By definition, mesenchymal stem cells are cell types that can differentiate into osteoblasts, fibroblasts, adipocytes, and chondrocytes (33).

Many mesenchymal stem cell sources are discarded on account of extraction difficulty, ethical concerns, and invasiveness (34). However, there are still several tissues from which mesenchymal stem cells can be extracted, such as bone marrow (35), adipose tissue (36), dental pulp (37), umbilical cord (38), placenta (39), peripheral blood (40), synovial fluid (41), endometrium (42), muscle (43), and skin (44).

Bone marrow, adipose tissue, and teeth are the most used tissue sources for mesenchymal stem cell extraction due to accessibility, initial quantity in the tissue and potential to generate bone, adipose and muscular tissue required for meat building. Nevertheless, only a few hundreds of mesenchymal cells can be obtained from these tissues (45), requiring a scaled-up process to produce the hundreds of millions of stem cells needed for cultivated meat production (46). Other characteristic properties of mesenchymal stem cells are their adherent growth under laboratory conditions (47) and a phenotype constituted by surface markers CD73, CD90, and CD105 (48), as well as the lack of CD45, CD34, CD14, CD11b, CD79a, CD19, and Class II histocompatibility complex antigens (49).

Additionally, mesenchymal stem cells grown in laboratory conditions are prone to differentiate spontaneously, causing a phenomenon called “loss of stemness” (50), which can be defined as the loss of differentiation capability and characteristic surface marker profile due to laboratory conditions.

Every connective tissue can be a source of mesenchymal stem cells, but many sources are discarded due to difficulties in extraction, ethical concerns, and invasiveness (34).

The main mesenchymal stem cell tissue sources are bone marrow (35), adipose tissue (36), dental pulp (37), umbilical cord (38), placenta (39), peripheral blood (40), synovial fluid (41), endometrium (42), muscle (43), and skin (44).

Bone marrow, adipose tissue, and teeth are the most used tissue source for mesenchymal stem cell extraction due to accessibility, initial quantity in the tissue and potential to generate bone, adipose and muscular tissue required for meat building.

The mesenchymal stem cell-based cultivated meat production process starts with the tissue extraction from animals (bone marrow, teeth, or adipose tissue), followed by the isolation of mesenchymal cells in a suitable medium containing nutrients and growth factors to allow full expansion of the cells from hundreds to hundreds of millions. Afterwards, the mesenchymal stem cells are differentiated to form muscular fibers that are finally integrated into a three-dimensional organization to form the final meat product.

The most important challenge for mesenchymal stem cell-based meat is the large-scale production of this cell type to produce mainly muscular fibers for meat production (51). The mesenchymal stem cells are present in hundreds within the original tissue (52) and the meat production require tens to hundreds of millions of stem cells (53).

This process is restricted by two main constraints: First, the genetic drift and “loss of stemness” which is commonly observed in laboratory mesenchymal stem cell culture. Second, the natural adherent growth, both post a challenge due to efficient relationship of space and cell growth surface.

This is caused by laboratory conditions not being the same growth conditions that the mesenchymal stem cells have within the body, posting a selective pressure to the cells. The more laboratory passages, the more different the cells will be from the original characteristics (54), both genetically and functionally (55).

In summary, mesenchymal stem cells are a suitable cell type for producing cultivated meat due to its ease of tissue collection and laboratory culture as well as the potential to generate fibroblasts, osteocytes, and chondrocytes as building blocks for cultivated meat.

However, the requirement to scale up the cell biomass is restricted by the low initial number of mesenchymal stem cells in the original tissues, the adherent nature of the cells and the loss of stemness in addition to genetic drift caused by long laboratory culture incubation.

Minimal handling and a short culture period are required to produce hundreds of millions of mesenchymal stem cells required for meat production. Through this review, the most commonly used solutions for these limitations will be broadly analyzed with the perspective of fulfilling the potential for cultivated meat production process implementation.

## Effects of laboratory culture in genetic drift and loss of stemness

Since the start of mammalian cell culture within laboratory, it has been reported that different types of cell lines from distinct sources have experienced certain grade of genetic drift: functional genomic changes within the same lines across passage numbers and laboratories (56).

A more concerning situation regarding genetic drift is shown in the reports of some cases of the formation of immortalized cell lines after routine passage of cells from primary cultures of different tissues (57) caused by the structural genomic mutations and expression changes (58).

The immortalization of cell lines is related to mutations in the genome causing activation of oncogenes. Those oncogenes promote deregulation of the cell cycle and deactivation of suppressor genes to inhibit cell apoptosis (59). For instance, in HaCAT cells, a non-tumoral human keratinocyte, the spontaneous immortalization is caused by the loss of Y-chromosome, short arm-loss of chromosome 3, 9, and 4 and partial trisomy of long arm of chromosome 9 (60).

In this context, one concerning report was published stating that HaSKpw cells, a primary culture keratinocyte cell line obtained from normal human adult skin, spontaneously developed a tumorigenic phenotype accompanied by a metastasis profile after just 22 passages (59).

This property of spontaneous immortalization in laboratory culture is more frequent in non-primate cell lines (61), which are the most common source of cells used as building blocks for cultivated meat production (32).

Laboratory environment and the sequential subcultures expose cells to a selective pressure that promotes the growth of cells that are capable to quickly adapt to the subculture process. This cell adaptation is mediated by genome-wide mutations that can cause problems in reproducibility, process standardization and lot to lot homogeneity.

However, genetic changes causing oncogene activation promoting the expression of a tumorigenic profile can cause health concerns in meat-product final consumers and require attention within the developing cultivated meat production industry. There must be a genetic quality control test for the cells used to manufacture cultivated meat to detect mutations within their genome with emphasis on oncogene activation mutations.

As a fair base to search tumorigenic profiles in stem cells, common human cancer mutation hotspots can be watched over as potential target sequences to detect oncogenic mutations in animal cells used for cultivated meat production as mentioned in Table 1.

**Table 1:** Potential mutational hotspots identified in human cancer cell lines to look up in animal cell-based cultivated meat production.

Mutational hotspot	Cancer cell lines	References
TP53	Lung/Breat/Colorrectal/Glioma/Liver/Pancreatic/Leukemia	Zonneville et al. (62)
KRAS	Lung/Colorrectal/Pancreatic/Leukemia	Bear et al. (63)
BRAF	Colorrectal/Melanoma/Leukemia	Berzero et al. (64)
PIK3CA	Breast/Colorrectal/Glioma/Leukemia	Yu et al. (65)
APC	Colorrectal/Melanoma/Pancreatic	Fodde (66)
BRCA1	Breast/Glioma/Liver	Drikos et al. (67)
ATM	Colorrectal/Glioma/Melanoma	Parenti et al. (68)
SMAD4	Colorrectal/Pancreatic	Li et al. (69)
MLL3 and MLL4	Lung/Melanoma	Dorighi et al. (70)
HNF1A	Glioma/Liver	Liu et al. (71)

Total RNA sequencing (72), flow cytometry (73), genome-wide sequencing (74), and quantitative PCR (75) are the four main techniques used to detect changes and activation of tumorigenic profiles in mesenchymal stem cells used for cultivated meat production.

The molecular technique selection depends on the depth and sensitivity required to detect these mutations with tumorigenic potential. Regulatory requirements need to be developed to decide the best practices for this issue while having a homogenous methodology to achieve this goal.

Additionally, laboratory conditions used to cultivate mesenchymal stem cells affect the functional properties and cause “loss of stemness” of these type of cells.

The most important characteristic for meat production is that mesenchymal stem cells preserve their ability to produce muscular, bone and adipose tissue to produce various meat products (76). This property can be lost with the continuous subculture under laboratory conditions: the more passages performed in the cells, the higher the probability that they lose their ability to differentiate into fibroblasts, osteocytes and adipocytes.

Genetic expression is crucially related to stemness preservation during mesenchymal stem cell culture. Wang et al. (77) have performed a genome-wide analysis of coding and non-coding RNAs from mesenchymal stem cells between Passages 4, 6, and 12; detecting significant changes in expression that are related to the decrease of proliferation, differentiation, and immunosuppression properties as a result of the number of passages.

These changes have been confirmed in protein proteomic profiles from human umbilical cord-derived mesenchymal stem cells from passages 3 to 10 (78), where significant changes in protein expression have been detected in each passage. Interestingly, passaging cells after 24–48 h caused the expression of multiple cancer genes.

This subculture periodicity is abnormal because mesenchymal stem cells are routinely subcultured twice a week (79). However, an accelerated assay that evaluates the effect of a large number of subcultures in the stemness and oncogene activation of mesenchymal stem cells can be performed, if needed. This type of experiment is really useful to draw conclusions on the long-term laboratory culture effects in cells within a fraction of the time it would routinely take, as subcultures are executed in a shorter periodicity than what is usually used.

The aforementioned expression changes are related to the loss of their property to generate muscular tissue and changes in the growth rate, which affect reproducibility of the general meat generation process. For this reason, a critical variable that must be determined during mesenchymal stem cell-based meat production is the interval of passage number where the cells preserve the property to generate muscular, adipose and bone tissue additionally considering that oncogene expression is not upregulated due to mutations in the genome.

The cryopreservation technique where cells are stored at ultracold temperatures to maintain optimal cell viability and functionality (80) has been proposed as a solution to avoid “loss of stemness” and genetic drift caused by a large number of laboratory culture passages. This technique has been widely used to stop cell proliferation, DNA replication and preserve mesenchymal stem cells from genetic drift and “loss of stemness” for a long-term period (21).

It has been demonstrated through freeze–thaw protein profiling from human umbilical cord-derived mesenchymal stem cells (78) that after 48 h of thawing, the cells recover their original protein profile. It was also discovered during these experiments that the miRNA profile expressed during the first 24 h after thawing resembles that of small lung cancer, hypertrophic and dilated cardiomyopathy (78).

With this information, cryopreservation is a great technique to limit the passage number and preserve the functionality, as it is only required to avoid using mesenchymal stem cells less than 48 h after thaw to avoid significant changes in expression profile.

Other additional aspects that can post difficulties in all stem cell applications in terms of reproducibility and standardization are the donor and the source of the mesenchymal stem cells (81).

These genetic, protein, and biological changes occurring in mesenchymal stem cells grown in laboratory post a challenge to determine the best practices for large-scale mesenchymal stem cell production keeping the differentiation properties during the process and avoiding the expression of cancer genetic profiles.

## Pharmaceutical-like quality control during cell production for cultivated meat?

For the safety, standardization, and reproducibility of mesenchymal stem cell usage in cultivated meat production, some quality control techniques currently used in the pharmaceutical industry for vaccine and drug-producing organisms can be proposed as guidelines for cell quality control within cultivated meat production processes.

Within the pharmaceutical industry, regulatory agencies such as the U.S. Food and Drug Administration (82) have proposed to test for tumorigenicity, oncogenicity, and genetic stability, as well as performing cell identification assays for mammalian cells used for biotech pharmaceutical drugs before the approval of the commercialization of recombinant proteins that these cells produce.

The FDA also requires that the producer cells are tested to detect any microbial contamination, with special emphasis on *Mycoplasma*, a common mammalian cell contaminant (83) as well as the presence of virus (84), purity of the cell culture (82) and a functional test (85). *Mycoplasma* and viral agents are common contaminants for mesenchymal stem cells (86), consequently, a microbial control of the cells must be executed emulating the ones performed in pharmaceutical industry.

For mesenchymal stem cells used for cultivated meat, a differentiation assay to test the ability of cells to generate muscular, adipose and bone tissue would be the most convenient functional test (87).

More specific quality controls are required to assure that the donor-based variability, such as age, source, season of sample recollection and laboratory passages, do not affect the final characteristics of the meat products. Multiple examples in pharmaceutical industry can be mentioned where the variability of monoclonal antibody production by different CHO cell lines can be significant (88). These differences can be even more significant in mesenchymal stem cells obtained from different donors and sources.

**Figure 2:**
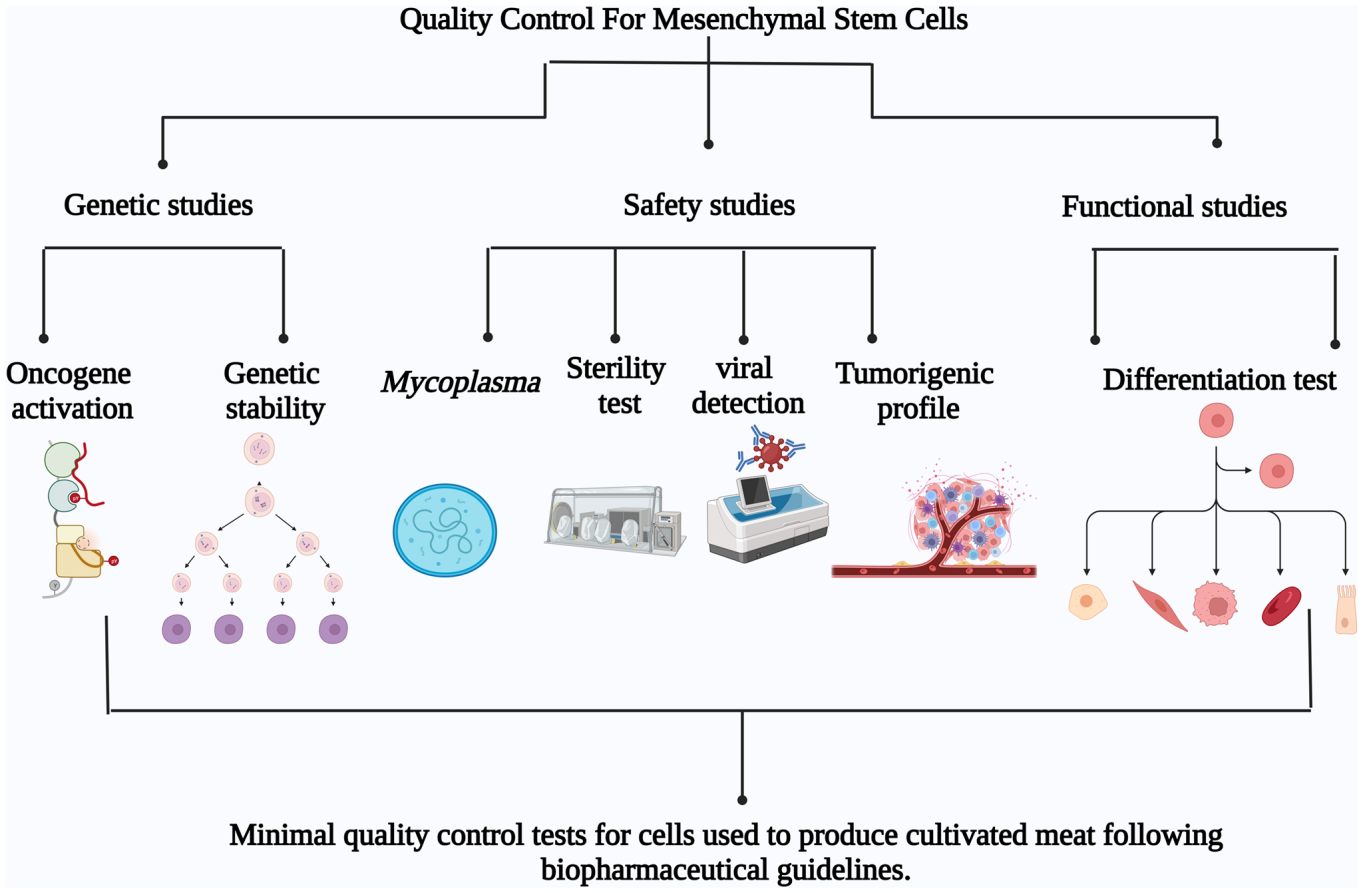
Minimum quality control tests for mesenchymal stem cells used for cultivated meat production based on pharmaceutical guidelines. The proposed tests can be divided in three main groups: Genetic studies (oncogene activation and genetic stability), Safety studies (*Mycoplasma* detection, Sterility test, Viral detection and Tumorigenic profile) and Functional studies (Differentiation test).

Minimum quality control tests for mesenchymal stem cells used in meat production must be determined to assure the standardization and safety of the use of mesenchymal stem cells. Based on pharmaceutical practices, genetic analysis to demonstrate genetic stability and lack of oncogene activation, *Mycoplasma* test, sterility test, viral contamination detection and tumorigenicity test to guarantee safety and cell health accompanied with functional assays such as differentiation test would be essential as quality controls to determine the maximum number of passages where the cells retain genetic stability, safety and functionality (Figure 2).

In conclusion, a large collaborative activity is required between food and health regulatory authority and cultivated meat experts to determine the quality control test required for cells in this industry, but pharmaceutical controls for drug producer cells can serve as a perfect first step to guide the effort.

This approach is deemed to be the most fruitful as both industries, pharmaceutical and cultivated meat industry, work with animal cells and similar techniques, so potential problems are shared, and the solutions proposed in pharmaceutical industry can be implemented with nearly the same regulatory success.

## Solving the issue of adherence in large scale stem cell culture for meat production

In addition to genetic drift and loss of “stemness,” adherent growth from mesenchymal stem cells posts an extra challenge for large-scale cultivated meat production.

Mesenchymal stem cells are plastic adherent spindle-shaped cells (89) that have anchorage dependence to grow. This property limits the surface area available for the cell growth related to the volume occupied by the culture container, which increases the costs and difficulty, along with the decrease of production efficiency (90).

**Figure 3:**
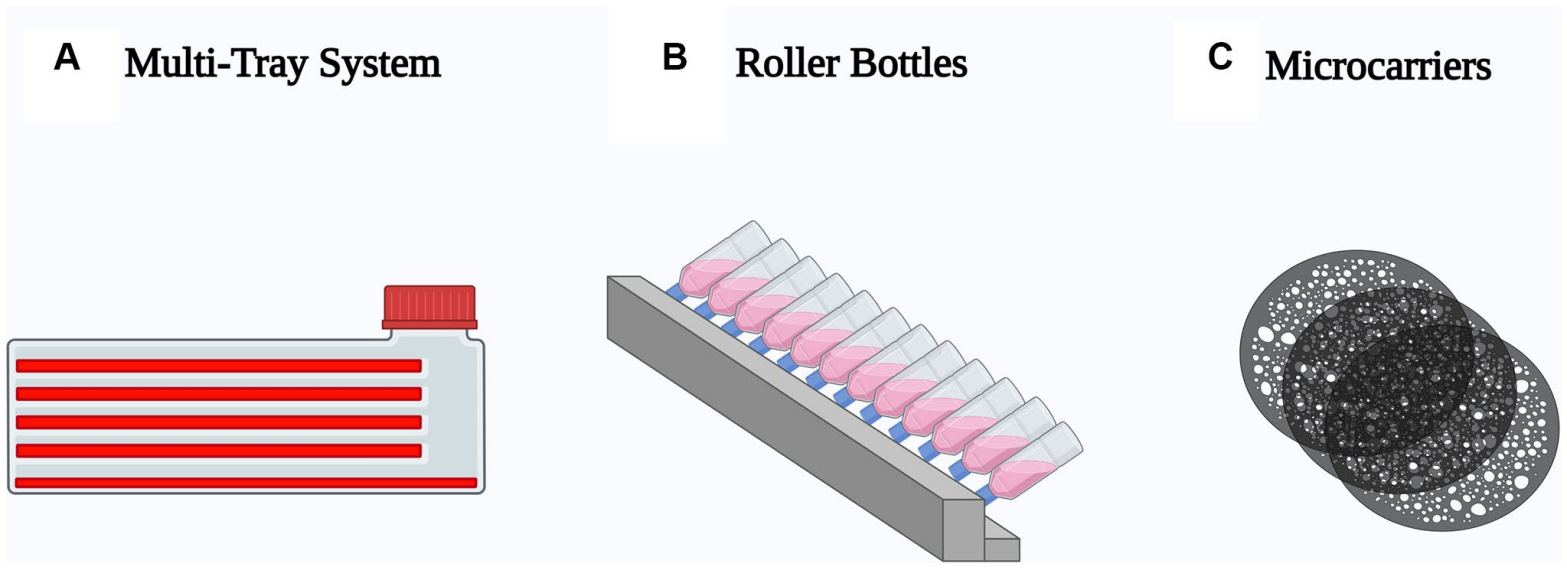
Schematic representation of the most used techniques to scale-up mesenchymal stem cell production. **(A)** Multi-tray system, optimized T-flasks with multiple stacked growth surfaces. **(B)** Roller bottles, cylindrical container incubated in a roller machine to enhance the growth surface. **(C)** Microcarriers, spherical particles suspended in agitated medium.

A variety of techniques currently employed for adherent cell expansion share similar characteristics, providing an increased surface area for cell adherence and proliferation, and a more efficient surface area to volume ratio to facilitate gas exchange. Three different approaches have been mainly used to scale-up mesenchymal stem cell production: multi-tray system, roller bottles and microcarriers (Figure 3).

The scale-up of mesenchymal stem cells using multi tray system consists of the use of T-flasks with an optimized height to support the growth areas for adherent cells, close enough to enhance the space occupied by the flasks within the incubator (91), expanding the cell production from 10 to 20 times.

Although this space optimization is a big advantage to support multi-tray system, the scalability is limited since the bigger the scale of the system and the number of cells required, the more difficult it is to manipulate and store the bottles. In addition, oxygen availability, the quality and reproducibility of the process are questionable (92).

This lack of reproducibility, specifically of the nutritional and metabolic requirements, complicates the multiplication and differentiation of stem cells into muscle fibers (93).

The structure of multi-tray systems is an obstacle to muscle cell differentiation because, in natural tissue, this process occurs through the blood vessels that transport nutrients by capillarity (94), but in multi-tray culture it occurs by direct (or close) contact of the cells with the oxygen-containing culture medium, which, among other things, limits the thickness of the growing tissue in front of cell attachment surfaces (91).

Multi-tray systems expand the growth surface for cell attachment but decrease oxygen availability, reproducibility, and structural support for the differentiation of muscle fibers for cultivated meat production. Additionally, the required scale up of cultivated meat production cannot be achieved without using large numbers of multi-tray systems. Multi-tray systems are not a solution toward achieving adherence of stem cells, to scale-up their production.

The roller flask technique was designed by Gey in 1933, with the aim of maintaining large numbers of cells at low cost using less culture medium (95). This technique has been widely used in the biotechnology industry, especially in relation to the development and production of vaccines (96). One of their main characteristics is that they are placed in a gas-tight chamber to avoid water loss in both the medium and the cells.

This type of technique is considered a monolayer culture and provides a larger surface area compared to standard T-flasks, making it ideal in terms of surface efficiency (97). One of the main differences compared to traditional T-flasks is that it allows the culture medium to be stirred homogeneously, which avoids the agglutination of gradients that can disturb cell development, as well as ensuring the development of a thin layer of medium covering the cells, allowing a global exchange of gasses (95).

The main disadvantages of roller bottles are the lack of industrial scalability because the bigger the cellular production, the bigger the cell surface required, which is related to a roller bottle diameter and a bigger empty space in the middle of the bottle or a bigger number of roller containers required (98). Additionally, the bioprocess conditions such as pH, dissolved oxygen and nutrient amount cannot be controlled (99).

Although multi-tray systems and roller bottles play an important role in mesenchymal stem cell scale-up, there is still a low efficiency with respect to surface area/volume ratio, which makes it less efficient for industrial scalability purposes and, consequently, for cultivated meat production.

In response to these limitations, the microcarrier methodology was first described by Van Wezel (100), who described a system by which anchorage-dependent cells could attach to and grow on small particles suspended in a shaking culture. Of the three potential solutions mentioned above, microcarriers offer an adhesive spherical surface that can efficiently optimize the surface to volume ratio (101).

The microcarriers are constituted by a polymeric nucleus formed by materials such as dextran, polystyrene, glass, cellulose, gelatin, collagen, alginate, or chitosan (102). The nucleus is covered by materials that promote cell adherence, such as collagen, poly-lysine, laminin, fibronectin, vitronectin, thrombospondins, tenascin, proteoglycans and glycosaminoglycans (103).

The foundation of this methodology is that cells attach to a microcarrier, a small particle, as they begin to develop into confluent growth, resulting in adherent cells growing in suspension within a stirred tank bioreactor, which allows for a greater scale-up than the other techniques mentioned above, as well as allowing for the potential ease of scalability, process monitoring and controllability that makes bioreactor culture commonplace in the biopharmaceutical field (104).

Another advantage from microcarriers is that these systems also require little human intervention once the process is underway, which reduces the risk of contamination (105). These microcarriers can be solid or porous, and the materials can be selected according to the intention of the culture and the cell type (106). The final step of the bioprocess is the separation of cultured cells from the microcarriers, where the cultured cells are used for downstream applications (107).

Cultured cells must be separated from microcarriers through the use of enzymes such as trypsin–EDTA, the microcarrier degradation or cleavage (108). This process can be complex, time-consuming and affecting the cell production efficiency (109).

To avoid the microcarrier cell separation in the meat production process, the use of edible materials and hydrogels is advised in the production of the microcarrier nucleus (110). With the use of this technique, the microcarriers with cells can be directly applied in the final meat product, decreasing downstream procedures and cell loss within the process.

The edible materials that can be used as the base material for microcarrier production are polysaccharides such as starch, alginate, carrageenan, chitosan, cellulose, carboxymethylcellulose, and pectin, and polypeptide treatment for cell attachment such as collagen, gelatin, and gluten (111).

Despite the great advantages offered by edible microcarriers, caution must be taken in the direct integration of these structures in the final meat product: a negative effect in the final organoleptic properties, toxicity caused by the cross-linking substance used to add molecules to the microcarriers for cell adhesion, and cooking and storage temperature (112).

Additionally, the microcarrier can develop a microenvironment to differentiate mesenchymal stem cells into fibroblasts with reproducibility (113). Nano-surface structures have been integrated to microcarriers with the objective of mimicking the final three-dimensional tissue structure (114).

The following Table 2 summarizes the advantages and disadvantages of the three scale-up techniques discussed in this review:

**Table 2:** Advantages and disadvantages of three animal cell scale-up techniques.

Parameter	Multi-tray System	Roller Bottle	Microcarrier cultures
Most growth surface area	More than the roller bottles, less than the microcarrier cultures	The least of the three	The best of the three
Increase of growth surface	Use more bottles	Use more bottles	Increase the microcarrier density or the bioreactor volume
Scale-up obstacles	The bigger the scale, the more intensive and complicated work needed	The bigger the scale, the more intensive work needed	The more difficult to maintain oxygen and nutrient homogenous diffusion
Media homogeneity	Variability within the multi-tray system	Homogenous	Homogenous
Cell homogeneity	Variability within the multi-tray system	Decrease as the bottle number increase	Homogenous
Continuous monitoring of bioprocess parameters (pH, dissolved oxygen, temperature)	Not monitored	Partially monitored	Completely monitored
Adjustment to bioprocess parameters (pH, dissolved oxygen, temperature)	Not controlled	Not controlled	Completely controlled
Work intensity to separate cells	Increase proportionally with the number of bottles used	Increase proportionally with the number of bottles used	Unitary operation that can be scaled-up easily
Possibility to avoid the cell separation step	Not possible	Not possible	Possible by using edible materials for the microcarriers
Exposed to shear forces	Not exposed	Not exposed	Exposed

After the comparison performed in Table 2, it can be concluded that microcarriers, with emphasis on edible microcarriers, are the most suitable scale-up technique for cultivated meat production process due to the efficient optimization of volume to surface ratio, the ease to grow adherent cells in a bioreactor accompanied with standardization ease, critical parameter control, the versatility of production volumes, and the decrease in downstream operations, resulting in the integration of edible microcarriers in the final meat product and the generation of a favorable microenvironment for muscle differentiation of mesenchymal stem cells.

## Conclusions and perspectives

Animal mesenchymal stem cell-based cultivated meat production is a promising solution to decrease the environmental impact that the traditional meat production process causes while maintaining the closest nutritional profile compared to other alternative sources such as plant and fungi that are currently commercially available.

The most important obstacles for large-scale cultivated meat production process implementation are the anchorage-dependent growth from mesenchymal stem cells and the genetic drift that causes loss of stemness and oncogene activation.

Genetic drift increase is highly related to the time in laboratory culture. This condition requires the determination of a limit number of passages for the stem cells supported with genetic and functional studies. This restriction assures that the cells can differentiate in a homogenous process in every passage and lack the expression of oncogenes while also decreasing the time available for the generation of the hundreds of millions of cells that are required for meat production.

Genetic, identity, microbiological and functional studies that resemble the ones performed to productor cells within the biopharmaceutical industry can give some guidance for the required analysis for the stem cells used for cultivated meat production to test their genetic stability and functionality with the objective of lot homogeneity, process standardization and customer safety.

Additionally, the anchorage-dependence from mesenchymal stem cells require them to grow adherently, limiting the scale-up operations to optimize the surface to operation volume to obtain the most growth area with the minimum of occupied space.

For this problem, microcarrier technology is the most promising solution because of its possibility to grow adherent cells in a bioreactor, the efficient volume to surface ratio optimization, and the wide research and development of new materials and structures for microcarrier production.

To address the challenges that were reviewed throughout this article, several “next steps” would have to be taken, namely: the standardization of microcarrier production following food and health regulation, the design and execution of the scale-up train for a large-scale bioreactor, the implementation of industrial scale mesenchymal stem cell production, the differentiation protocol to form muscular tissue from stem cells grown on the microcarriers, the definition and execution of downstream operations, and the development of edible microcarrier based-cultivated meat formulations.

In conclusion, nowadays it is clear at laboratory scale that edible microcarriers are the most promising tool for the industrial production of cultivated meat. Now, testing the potential at industrial scale and designing the unitary operations to be performed after the bioreactor-based stem cell production are the next steps scientists should focus on in order to achieve large-scale production of cultivated meat.

## Author contributions

MJ-R and RC-S devised the review paper, the main conceptual ideas and proof outline. MJ-R, AC-H, and LR-V made the systematic review and wrote the first draft of the article. MJ-R, JP-S, and RC-S worked in editing and reviewing of paper. All authors contributed to the article and approved the submitted version.

## Funding

This research was supported by Tecnológico de Monterrey, Campus Monterrey and for the scholarship (CONACYT CVU-456151) of MJ-R. Also, it is supported by Challenge-Based Research Funding Program at Tecnológico de Monterrey, since this program funded the entire project in the grant with number E004 - EIC-GI01 - A-T23 - D.

## Conflict of interest

The authors declare that the research was conducted in the absence of any commercial or financial relationships that could be construed as a potential conflict of interest.

## Publisher’s note

All claims expressed in this article are solely those of the authors and do not necessarily represent those of their affiliated organizations, or those of the publisher, the editors and the reviewers. Any product that may be evaluated in this article, or claim that may be made by its manufacturer, is not guaranteed or endorsed by the publisher.
